# OP49 Building Capacity In Horizon Scanning: Developing A Tool For Data Extraction And Search Recommendations

**DOI:** 10.1017/S0266462325101074

**Published:** 2025-12-29

**Authors:** Hannah O’Keefe, Sonia Garcia Gonzalez-Moral, Chris Marshall

## Abstract

**Introduction:**

Horizon scanning is a methodology used to identify signals and insights that serve as early awareness indicators. We present a free web application that houses the National Institute for Health and Care Research (NIHR) Innovation Observatory’s data dictionary, allowing users to generate a data extraction template and provide search source suggestions. This resource aims to build capacity in horizon scanning by promoting knowledge exchange and improving productivity.

**Methods:**

We identified core data extraction points from four years of horizon scans (n=20). These were divided into 11 categories and linked to scan type, time horizon, and potential search sources. We used Microsoft Access to create a relational database, later converted to SQLite. Python and Streamlit were used to develop a front-end web application. The tool enables users to select parameters relevant to their horizon scan needs and generates customized data extraction templates and search source suggestions. Eight data points specific to the NIHR Innovation Observatory were included in the data dictionary but excluded from the template generator.

**Results:**

The web application incorporates 148 data points linked to nine types of horizon scans, three scan categories, three time horizons, and 183 search sources. Users can generate tailored data extraction templates based on selected criteria, facilitating a structured approach to horizon scanning. The tool’s features include an intuitive interface, customizable templates, and comprehensive search source recommendations. While formal evaluation is planned, initial internal feedback indicated the tool effectively reduces time on tasks and improves productivity. Future updates will focus on incorporating user feedback and enhancing functionality.

**Conclusions:**

This free-to-access resource provides a starting point for anyone wanting to conduct a horizon scan. It offers a comprehensive data dictionary, promotes knowledge exchange and transparency, and allows users to generate robust and reproducible data extraction templates. Plans are underway to formally evaluate the tool’s effectiveness and gather user feedback to inform future updates.

